# Hafnium oxide nanoparticles: toward an *in vitro* predictive biological effect?

**DOI:** 10.1186/1748-717X-9-150

**Published:** 2014-06-30

**Authors:** Julie Marill, Naeemunnisa Mohamed Anesary, Ping Zhang, Sonia Vivet, Elsa Borghi, Laurent Levy, Agnes Pottier

**Affiliations:** 1 Nanobiotix, 60 rue de wattignies, 75012 Paris, France

## Abstract

**Background:**

Hafnium oxide, NBTXR3 nanoparticles were designed for high dose energy deposition within cancer cells when exposed to ionizing radiation. The purpose of this study was to assess the possibility of predicting *in vitro* the biological effect of NBTXR3 nanoparticles when exposed to ionizing radiation.

**Methods:**

Cellular uptake of NBTXR3 nanoparticles was assessed in a panel of human cancer cell lines (radioresistant and radiosensitive) by transmission electron microscopy. The radioenhancement of NBTXR3 nanoparticles was measured by the clonogenic survival assay.

**Results:**

NBTXR3 nanoparticles were taken up by cells in a concentration dependent manner, forming clusters in the cytoplasm. Differential nanoparticle uptake was observed between epithelial and mesenchymal or glioblastoma cell lines. The dose enhancement factor increased with increase NBTXR3 nanoparticle concentration and radiation dose. Beyond a minimum number of clusters per cell, the radioenhancement of NBTXR3 nanoparticles could be estimated from the radiation dose delivered and the radiosensitivity of the cancer cell lines.

**Conclusions:**

Our preliminary results suggest a predictable *in vitro* biological effect of NBTXR3 nanoparticles exposed to ionizing radiation.

## Background

Ionizing radiation is a universal killer with ability to generate double strand breaks in the DNA; as such it is the principle target for cancer cell killing. Ionizing radiation-induced damage correlates with the energy dose deposition in each biological structure [1]. As it is well-known radiation always affects tissues surrounding the targeted tumor and most often the dose, effective to control the tumor, cannot be delivered due to damage to the surrounding healthy tissues.

Recently, nanotechnologies have paved the way to new approaches in local cancer therapy. Nanoparticles with high electron density offer the possibility to deposit high amounts of energy within the cancer cells when activated by ionizing radiation, providing appropriate bioavailability and persistence [2,3].

Nanocrystals of hafnium oxide, NBTXR3 nanoparticles, were designed for efficient cancer cell uptake and interaction with different types of ionizing radiation. The high density material at the nanoscale and ionizing radiation interactions render feasible the physical mode of action of radiotherapy directly from within the cancer cells.

Using Monte Carlo calculation, activation of NBTXR3 nanoparticles with high energy photons (1 or 6 MeV) already demonstrated a local energy deposit in specific subcellular structures containing the nanoparticles [2]. Advancing this concept, we have implemented a global *in vitro* research program to investigate if we could predict the biological response of cells treated with NBTXR3 nanoparticles exposed to ionizing radiation. First, we estimated the nanoparticle uptake in a panel of human cancer cell lines with increased nanoparticle concentration. Subsequently the NBTXR3 nanoparticle’s effect on cell survival was evaluated using the clonogenic survival response curve. Our results suggest that above a minimum number of nanoparticles per cell, the radioenhancement of NBTXR3 nanoparticles could be predicted from the radiation dose delivered and the intrinsic radiosensitivity of the cancer cell line.

## Methods

### NBTXR3 nanoparticles

NBTXR3 nanoparticles are functionalized hafnium oxide (HfO_2_) crystallites, bearing a marked negative surface charge in aqueous solution at pH 6-8. Specifically, the studies reported in the present article were performed with spherical nanoparticles with size and surface charge of approximately 50 nm and -50 mV respectively (see Additional file 1). NBTXR3 was used at increasing nanoparticle concentration from 50 μM up to 1600 μM.

### Human cancer cell lines and culture conditions

The radiosensitive cell lines HCT 116 (colorectal carcinoma) and Hs913T (fibrosarcoma) were purchased from the American Type Culture Collection (LGC Promochem, Molsheim, France) and the Interlab Cell Line Collection (Genova, Italy) respectively.

The radioresistant cell lines HT-29 (colorectal adenocarcinoma), FaDu (pharinx squamous cell carcinoma), HT-1080 (fibrosarcoma) and PANC-1 (pancreas epithelial carcinoma), were purchased from the American Type Culture Collection (LGC). The radioresistant cell lines NCI-H460-luc2 (non small cell lung adenocarcinoma) and CAL-33 (human head and neck squamous cell carcinoma) were purchased from Caliper life science (Villepinte, France). The radioresistant cell line 42-MG-BA (glioblastoma multiforme), was purchased from the Deutsche Sammlung von Mikroorganismen und Zelkulturen GmbH German Collection of Microorganism and Cell Cultures (Braunschweig, Germany).

Cells were grown according to each manufacturer’s recommendations (see Additional file 2). All cells lines were kept in an incubator at 37°C under a 5% CO_2_ humidified atmosphere.

### Cellular uptake of NBTXR3 nanoparticle by Transmission Electron Microscopy (TEM)

Cells were seeded into 6-well plates (in duplicate) at the density of 20 000 cells/cm^2^. NBTXR3 nanoparticle concentrations from typically 50 μM up to 1600 μM were tested. When cells were attached to the plate, NBTXR3 was added overnight (12 h-16 h) to the cells. Cells were then pre-fixed with 2.5% glutaraldehyde in cacodylate 0.1 M at pH 7.4 (Sigma Aldrich, Saint Quentin Fallavier, France) for 2 hours. Cells were subsequently post-fixed with 1% osmium tetroxide (Sigma Aldrich) for 45 minutes, washed and dehydrated in graded concentrations of ethanol (50%, 70%, 95% and 100%) (Sigma Aldrich). Cell samples were embedded with EPON (Oxford Instruments, Gometz la Ville, France). The resin sample block was trimmed, 90 μm thin-sectioned to thickness of 70 nm, (diatome diamond knives, Euromedex, Souffelweyersheim, France), and collected on grids (Euromedex) for observation under transmission electron microscopy (Zeiss, Omega 912, Service de Microscopie Electronique, IFR-83, Institut de Biologie Integrative, UPMC, Paris, France). For each cell line, a minimum of 20 cells were observed to estimate NBTXR3 nanoparticles cell uptake. Parameters evaluated included cluster number per cell and the cluster size. Size was split between “small cluster” with a medium of size around 0.45 μm of diameter and “large cluster” with a diameter around 1.8 μm.

### Evaluation of NBTXR3 nanoparticle effect on cells survival by the clonogenic survival assay

Cells were seeded into 6-well plates (in triplicate) within the range of 200 to 8000 cells/well. NBTXR3 nanoparticles concentrations from typically 50 μM up to 800 μM were tested. When cells were attached to the plate, NBTXR3 was added overnight (12 h-16 h) to the cells before delivering the radiation dose. The cells were cultured with antibiotics (penicillin/streptomycin, Invitrogen) from 10 to 15 days at 37°C under a 5% CO_2_ humidified atmosphere, allowing cells to form colonies. The colonies were fixed and stained with crystal violet solution (Sigma Aldrich) and individual colonies were counted using the standard procedure [4]. The Dose Enhancement Factor (DEF) was calculated for each radiation dose and each NBTXR3 nanoparticle concentration by taking the ratio of the surviving fractions of cells exposed to radiation (no NBTXR3 treatment) and of cells treated with NBTXR3 and exposed to radiation. Three (3) independent experiments performed in triplicate were pooled to calculate a mean surviving fraction and standard deviation (SF mean ± SD (*n* = 3)) and mean Dose Enhancement Factor (DEF mean ± SD (*n* = 3)).

### Cell survival analysis

Cell survival curves were obtained by analysing the mean surviving fractions (SF mean) with the linear quadratic (LQ) model (Equation 1) using the GraphPad Prism® V5.0 Software [5].

(1) $$S=e^{\left[‒\left(\mathrm{\alpha D}+{\mathrm{\beta D}}^{2}\right)\right]}$$

*S* represents the survival after radiation dose (D). α (Gy^−1^) and β (Gy^−2^) are the linear and the quadratic parameters respectively.

### Irradiation conditions

X-ray irradiations were delivered at dose rate of 1.26 Gy min^−1^ using X-Ray generator (200 kV, 15 mA, 0.2 mm Copper filtration, Institut Gustave Roussy, Villejuif, France). The 6-well plates were irradiated with a single dose (0 Gy (sham control) up to 4 Gy or 6 Gy) after incubation with NBTXR3, and then replaced into the incubator until colonies formed.

## Results

### In vitro exploration of NBTXR3 nanoparticle uptake in human cancer cell lines

**Figure 1:**
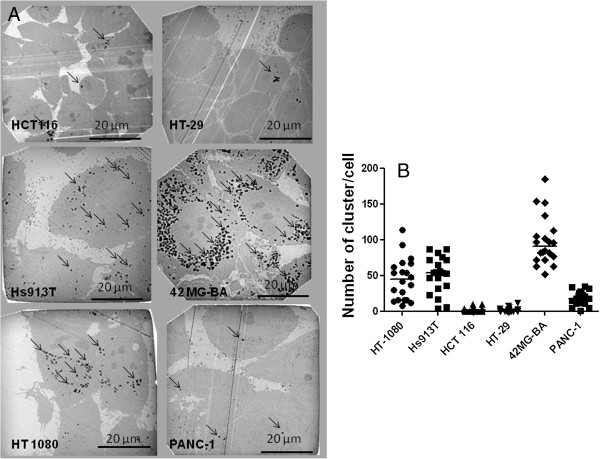
**Semi quantitative analysis of NBTXR3 nanoparticles cellular uptake.** Cells were treated with NBTXR3 overnight. **(A)** representative TEM images (NBTXR3 400 μM) for mesenchymal (Hs913T, HT-1080), epithelial (HCT 116, HT-29, PANC-1) and glioblastoma (42-MG-BA) cells showing clusters of nanoparticles in endosomes (black arrows) and **(B)** scatter plot and median value presenting the number of NBTXR3 nanoparticles’ clusters per cell. Scale bars = 20 μm.

**Figure 2:**
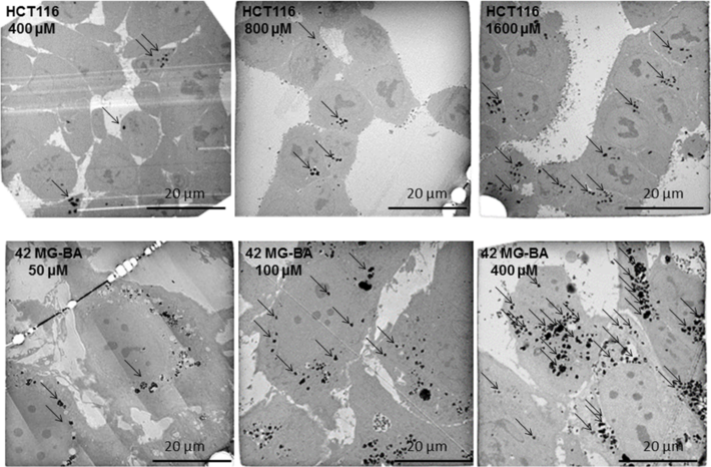
**NBTXR3 nanoparticles cellular uptake with increase of the NBTXR3 nanoparticle concentration.** Cells were treated with increasing doses of NBTXR3. Representative TEM images of HCT 116 cells treated with NBTXR3 400, 800 and 1600 μM and 42-MG-BA cells treated with NBTXR3 50, 100 and 400 μM. Scale bars = 20 μm.

**Figure 3:**
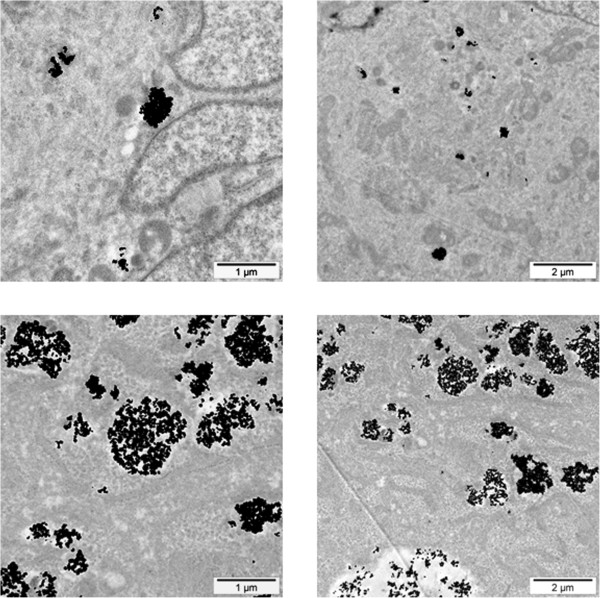
**Size of NBTXR3 nanoparticles’ clusters.** Representative TEM images of NBTXR3 clusters in PANC-1 (Top panel) and 42-MG-BA cells (Bottom panel) treated with NBTXR3 100 μM. Scale bars = 1 μm (left panel) and 2 μm (right panel).

The cellular uptake of NBTXR3 nanoparticle at 400 μM by epithelial (HCT 116, HT-29, PANC-1), mesenchymal (Hs913T, HT-1080) and glioblastoma 42-MG-BA cell lines was investigated by TEM (Figure 1A). NBTXR3 nanoparticles have been shown to interact with cellular membranes and to be taken up by cells (data not shown). Here, TEM images showed NBTXR3 nanoparticles forming clusters in the cytoplasm. Besides, the level of NBTXR3 nanoparticles taken up by cells depends on the specific cell line. In the panel of human cancer cell lines tested, mesenchymal and glioblastoma cell lines seemed more likely to uptake NBTXR3 nanoparticles when compared to epithelial cell lines. Figure 1B presents the number of clusters of NBTXR3 nanoparticles per cell for NBTXR3 concentration of 400 μM in HCT 116, HT-29, PANC-1, HT-1080, Hs913T and 42-MG-BA cell lines. The number of clusters varied between 8 and 114 (median 45), 4 and 87 (median 54), 63 and 185 (median 90) for the mesenchymal HT-1080 and Hs913T and the glioblastoma 42-MG-BA cell lines respectively. Clusters of NBTXR3 nanoparticles were found in every cell observed. In contrast, the number of clusters varied between 0 and 10 (median 2), 0 and 12 (median 0.5), 1 and 37 (median 18.5), for HCT 116, HT-29 and PANC-1 epithelial cell lines respectively.TEM images of the epithelial HCT 116 and glioblastoma 42-MG-BA cell lines, treated with increasing doses of NBTXR3 are presented in Figure 2. The number of clusters of NBTXR3 nanoparticles per cell increased with increase of the NBTXR3 nanoparticle concentration. Furthermore, the size of NBTXR3 nanoparticles clusters varied depending on the cancer cell line; as illustrated in Figure 3, a larger size of clusters is observed for the the glioblastoma 42-MG-BA model when compared with the epithelial PANC-1 model.

### In vitro assessment of NBTXR3 radioenhancement in a panel of human cancer cell lines

**Figure 4:**
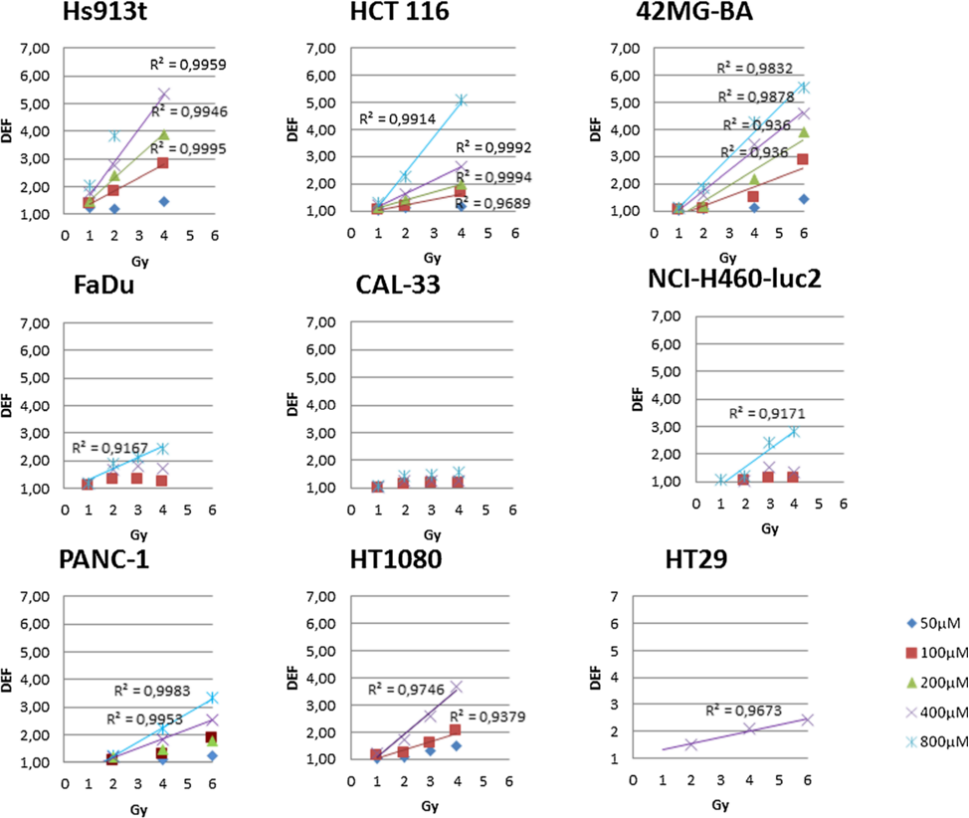
***In vitro *assessment of NBTXR3 nanoparticles radioenhancement.** Human cancer cells were treated with increasing concentrations of NBTXR3 nanoparticles overnight before being exposed to irradiation. Symbols represent the mean DEF. The data (mean DEF) were fitted by a simple linear regression.

**Figure 5:**
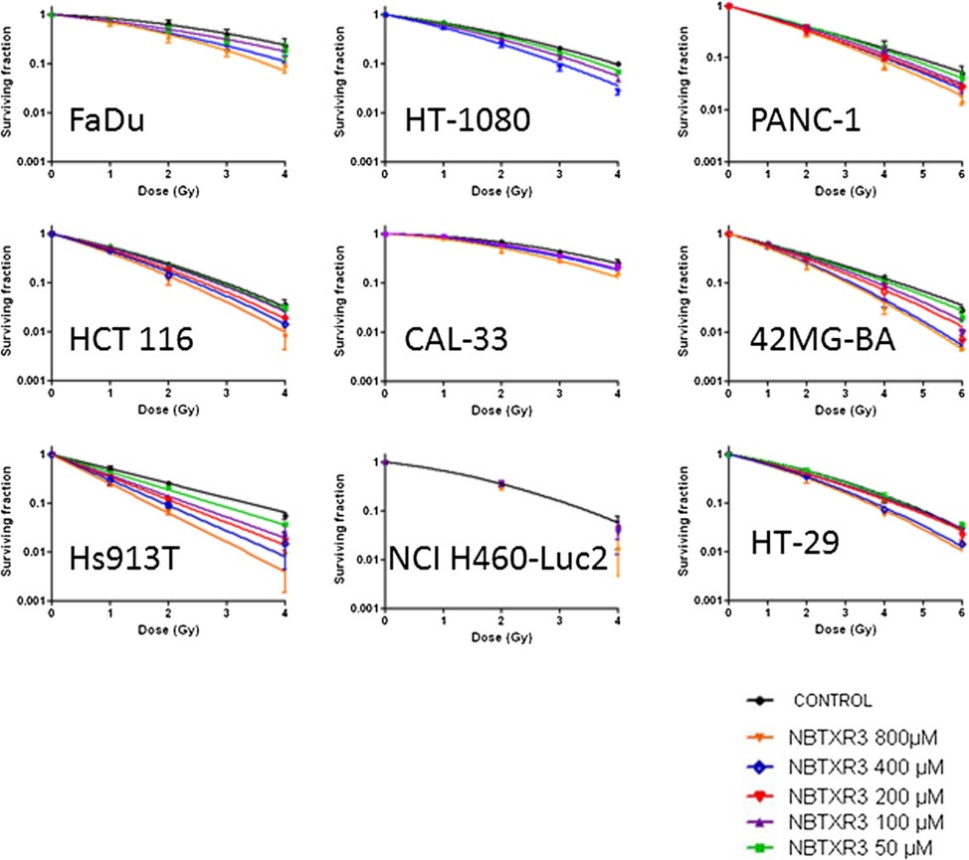
**Cell survival curves.** Human cancer cells were treated with increasing concentration of NBTXR3 nanoparticles overnight before being exposed to radiation. Symbols represent mean surviving fraction (mean SF ± SD). The data (mean SF) were fitted with the LQ model equation to estimate the α and β parameters.

Epithelial, mesenchymal and glioblastoma cancer cells, (radiosensitive and radioresistant), were selected to evaluate the surviving fraction and DEF of cells treated with NBTXR3 at increasing concentration and exposed to ionizing radiation (Figure 4 and Figure 5). The plating efficiency of tested models was always superior to 20% (data not shown). No clonogenic toxicity was observed under continuous incubation with NBTXR3 at increasing concentration in most cell lines in the panel. However, NBTXR3 at 300 μM and 800 μM showed IC_50_ in HT-1080 and Hs913T cells lines respectively (data not shown).

**Figure 6:**
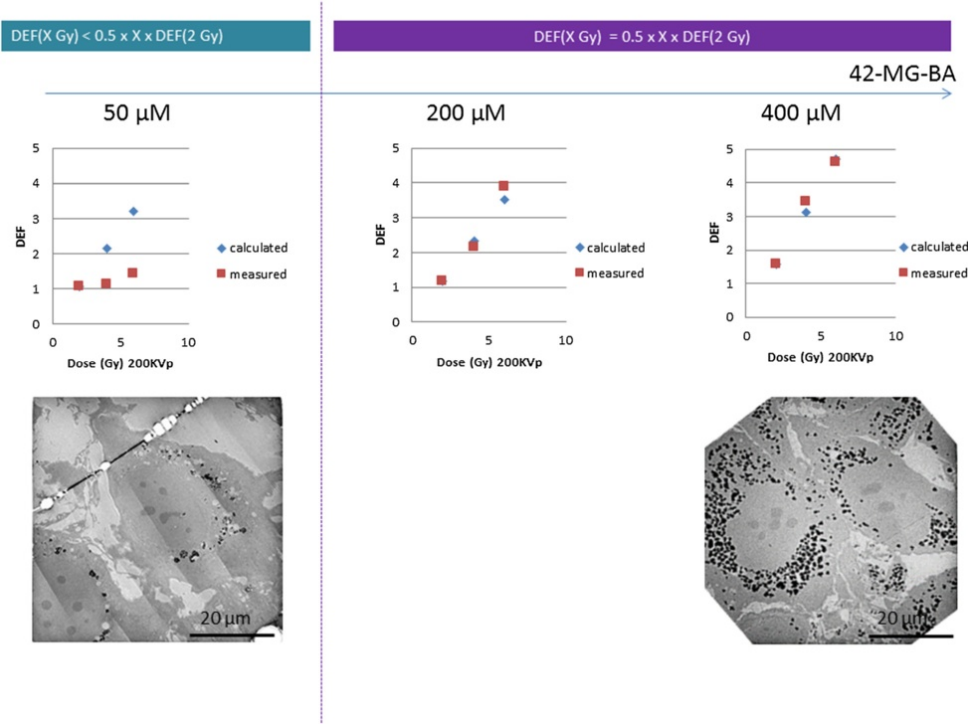
**Toward a predictive NBTXR3 nanoparticle radioenhancement.** Calculated and measured DEF values in 42-MG-BA cell line with increase NBTXR3 nanoparticles concentration and radiation dose and corresponding TEM images of NBTXR3 cell uptake. Scale bars = 20 μm.

Since, we believe the mode of action of NBTXR3 nanoparticles is to enhance dose rather than to sensitise cells. Therefore we do not consider the term radiosensitiser is appropriate in this situation. Analysis of the clonogenic survival data shows that the DEF is a function of dose. In order to analyse this dose dependence we treat the DEF measurements in a slightly unusual way. Rather than expressing the DEF at a given survival fraction, e.g. DEF_50_, we determined the DEF as a ratio of surviving fractions for a given radiation dose, i.e. for a given radiation dose (D) we determined a survival fraction in the presence of NBTXR3 nanoparticles, SF^+NP^(D). We then determined for the same given radiation dose (D) the survival fraction in the controls (i.e. without nanoparticles), i.e. SF^-NP^(D). For a given radiation dose, the DEF value was obtained by taking the ratio of the two survival fractions, i.e. DEF = SF^-NP^(D)/SF^+NP^(D).In the epithelial models (HCT 116, HT-29, PANC-1, NCI H460-luc2), the measured mean DEF values (over the triplicate measurements) ranged between 1.05 ≤ DEF ≤ 1.64 and 1.25 ≤ DEF ≤ 2.63 for NBTXR3 (400 μM) activated by 2 or 4 Gy respectively. NBTXR3 at 800 μM showed 1.21 ≤ DEF ≤ 2.24 and 1.57 ≤ DEF ≤ 5.09, activated by 2 or 4 Gy respectively. For the mesenchymal and glioblastoma cell lines (HT-1080, Hs913T, 42-MG-BA), the DEF values for NBTXR3 (400 μM) activated by 2 or 4 Gy ranged between 1.57 ≤ DEF ≤ 2.75 and 3.44 ≤ DEF ≤ 5.36 respectively. The DEF values increased according to the NBTXR3 concentrations and radiation dose (Figure 4). For most of the tested cell lines the DEF was found to increase linearly with NBTXR3 concentration and radiation dose. Moreover, for the epithelial cancer cell lines HCT 116, NCI-H460-luc2, PANC-1, the mesenchymal Hs913T, HT-1080, and the glioblastoma 42-MG-BA cancer cell lines, above a definite NBTXR3 concentration and radiation dose of 2 Gy, the DEF values were estimated using the equation 2 (Figure 6).

(2) $$\mathrm{DEF}\left(\mathrm{XGy}\right)=0.5\times X\times \mathrm{DEF}\left(2\mathrm{Gy}\right)$$

**Figure 7:**
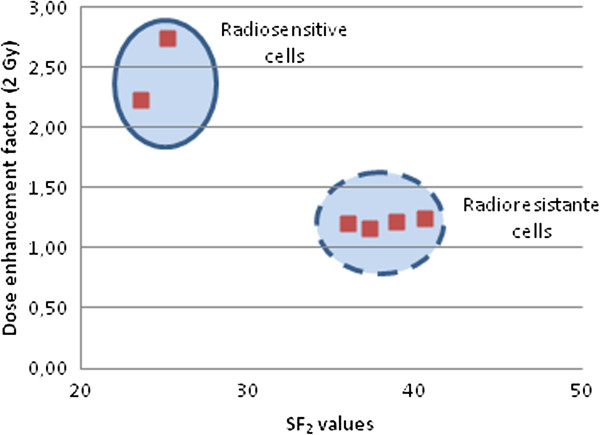
**Mean DEF at 2 Gy as a function of cell line radiosensitivity.** The reported mean DEF values at 2 Gy correspond to the onset of NBTXR3 concentration where equation 2 predicts an observable effect, and depend of the intrinsic radiosensitivity (SF_2_) of the cell line, see Table 1.

Equation 2 was applicable (*i.e.* the error between calculated and observed DEF was less than 20%) for NBTXR3 concentration ≥ 800 μM in the epithelial cancer cells lines. Meanwhile, this equation was applicable at lower nanoparticle concentrations, typically between 100 μM and 400 μM, in the mesenchymal and glioblastoma cancer cell lines (Table 1 and Figure 6). In addition, when equation 2 was applicable, the DEF value observed at 2 Gy was correlated to the intrinsic radiosensitivity (SF_2_) of the cell line (Table 1 and Figure 7).

**Table 1:** Toward a predictive NBTXR3 nanoparticle radioenhancement

		**Onset of NBTXR3 nanoparticles concentration where equation 2 predicts an observable effect**	
**Cell line**	**SF**_**2 **_**(mean ± SD)**	**[NBTXR3] μM (Onset)**	**DEF (2 Gy)**
HCT 116	22.4 (±9.14)	800	2.2 (±0.55)
Hs913T	25.2 (±2.27)	400	2.8 (±0.36)
NCI H460-Luc2	36.0 (±3.2)	800	1.2 (±0.06)
42-MG-BA	37.3 (±2.53)	200	1.2 (±0.03)
HT-1080	38.9 (±1.39)	100	1.2 (±0.06)
PANC-1	40.6 (±6.64)	800	1.3 (±0.25)
CAL-33	64.8 (±2.12)	nd	nd
FaDu	66.0 (±6.78)	nd	nd

Onset of NBTXR3 nanoparticles concentrations where equation 2 predicts an observable effect are presented together with the corresponding measured mean DEF values at 2 Gy. nd: non determined.

### Effect of NBTXR3 nanoparticles on the biological effective dose

The parameters associated with the survival curves are summarized in Table 2. For each cell line tested, the linear parameter α increased with increased nanoparticle concentration. For control cell lines (radiation alone), α ranged between 0.05 Gy^−1^ and 0.68 Gy^−1^ and reflected the radiosensitivity of each cell line (see SF_2_ values), whereas cells treated with NBTXR3 at 400 μM and exposed to ionizing radiation, alpha ranged between 0.43 Gy^−1^ and 0.74 Gy^−1^. The quadratic coefficient β, for both controls and NBTXR3 treated cell lines, ranged between 0.01 Gy^−2^ and 0.09 Gy^−2^.

**Table 2:** Radiobiological parameters for each cell line exposed to radiation with increased concentration of nanoparticles

**Cell line**	**SF**_**2 **_**values (mean ± SD)**	**Control**		**[NBTXR3]**									
				**50 μM**		**100 μM**		**200 μM**		**400 μM**		**800 μM**	
		**α**	**β**	**α**	**β**	**α**	**β**	**α**	**β**	**α**	**β**	**α**	**β**
42-MG-BA	37.3 (±2.53)	0.45	0.02	0.47	0.02	0.48	0.03	0.54	0.03	0.57	0.05	0.60	0.05
HCT 116	22.4 (±9.14)	0.54	0.08†	0.58	0.08†	0.60	0.08†	0.68	0.08†	0.74	0.08†	0.84	0.08†
PANC-1	40.6 (±6.64)	0.43	0.01	0.41	0.02	0.44	0.02	0.50	0.01	0.50	0.02†	0.51	0.02†
HT-1080	38.9 (±1.39)	0.33	0.06	0.37	0.07	0.44	0.08	nd	nd	0.55	0.07	nd	nd
Hs913T	25.2 (±2.27)	0.68		0.83		0.99		1.07		1.2		1.38	
HT-29	54.1 (±9.50)	0.28	0.05	0.30	0.05	0.39	0.03	0.40	0.03	0.43	0.05†	0.46	0.05†
FaDu	66.0 (±6.78)	0.12	0.06	nd	nd	0.26	0.04	nd	nd	0.31	0.06	0.28	0.09
CAL-33	64.8 (±2.12)	0.05	0.08	nd	nd	0.08	0.08	nd	nd	0.16	0.07	0.16	0.09

The LQ model was used to fit the surviving fraction data using GraphPad Prism® V5.0 Software, extracting α and β parameters. ^†^: β value was constrained to improve the quality of the fit. The SF_2_ value for each cell lines is reported.

**Figure 8:**
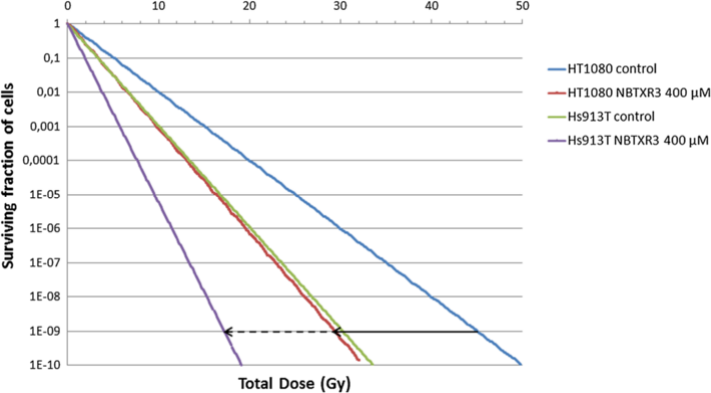
**“Effective” survival curve.** An effective survival curve was established for radioresistant HT-1080 and Hs913T cell lines, in the context of fractionated radiotherapy, 2 Gy per fraction, delivered once daily for 25 days, with presence of NBTXR3 nanoparticles (400 μM), and compared with radiotherapy alone control (arrow indicate the BED).

We established an effective survival curve model in the context of fractionated radiotherapy (2 Gy) once daily, for 25 days, for the fibrosarcoma cell lines exposed to 400 μM of NBTXR3, and compared the results of this model to corresponding predictions for radiotherapy alone. We assumed that the window between successive fractions has allowed the physiological repair (sublethal damage) in cells. The α and β parameters using the LQ model for the HT-1080 and Hs913T cell lines with NBTXR3 concentration of 400 μM and using the X-ray generator 200 kVp, were used in this simulation (Figure 8). From these data, it could be calculated that a total dose of approximately 29 Gy and 17 Gy was necessary to eradicate 10^9^ HT-1080 and Hs913T cells respectively (which is about equivalent to the number of cells in a tumor of approximately 1 cm^3^) with presence of NBTXR3 nanoparticles, whereas a total dose of approximately 45 Gy and 30.5 Gy respectively was necessary for the same outcome in these cell lines with radiotherapy alone.

## Discussion

### Uptake of NBTXR3 by cancer cells

Cellular uptake of nanoparticles depends on many factors, including nanoparticles size, shape, and composition of the protein corona [6-10]. Some studies have reported saturation of nanoparticle uptake within hours [11], whereas others after several days [12].

Kim *et al.* showed that uptake of nanoparticles by cells was influenced by the cell cycle phase with quantities of nanoparticles per cell being ranked according to G2/M > S > G0/G1 [13].

The cellular uptake of plasma membrane receptors mediated by clathrin is used by all known eukaryotic cells and is involved in many key cellular functions [14]. The pathway is exploited by toxins, viruses and bacteria to effect entry into cells. In epithelial cells, the size of clathrin-coated vesicles has been found to depend on the size of its cargo [15] with an observed upper limit of about 200 nm for virus uptake [16]. In mesenchymal stem cells, Huang *et al.* showed that phagocytosis and pinocytosis were the main courses of endocytosis for non-specific uptake of nanoparticles [17]. Also, many cancer cells (the most aggressive) have been described with phagocytic properties both *in vitro* and *in vivo*[18].

Here, we presented a clear trend for differential NBTXR3 nanoparticles’ uptake between epithelial and mesenchymal or glioblastoma cell lines, both in terms of number and size of clusters per cell. Extensive studies have already revealed differential NBTXR3 nanoparticles uptake according to the specificity of the cell line, for instance, fibrosarcoma cells were shown to capture more than fibroblast cells even if they share the same cell origin (data not shown). The exact mechanism for this differential uptake deserves more studies and is beyond the objectives of this paper.

### Effect of NBTXR3 nanoparticles on the biological effective dose

Radiation dose deposit within any tissue sample is linked to its ability to absorb/interact with x-rays. Introduction of material with a high electron density into the x-ray pathway can increase absorption as compared with water. Only the use of such material at the nanoscale can bring the physical mode of action of radiotherapy from within the cancer cells. Local dosimetry at the cellular level has yielded evidence of increased deposit of energy in specific subcellular structures containing the high electron density nanoparticles, *via* secondary electron emission (photoelectric and/or Compton effect), photon emission and also possibly *via* characteristic x-ray photon, Auger cascade and subsequent radical production. In addition, significant *in vitro* radioenhancement has already been reported to be related to the nanoparticles location at the subcellular level [2,3,19].

The Linear Quadratic model assumes that lethal radiation damage, as a result of double-strand breaks in the DNA, is created either as a consequence of a single ionizing event or as a consequence of two separate sublethal ionizing events that interact pairwise to create lethal damage [20]. α and β parameters are considered to be measures of the probability of inducing damage following a single or two separate sublethal ionizing events respectively [4,21]. In the panel of human cancer cell lines tested, the survival curves fitted using the LQ model showed that the α and not the β parameter, was systematically increased in cells treated with increasing concentrations of NBTXR3 exposed to 200 kVp photon beam, when compared to the radiation control (Table 2). These findings confirmed the expectation that higher energy dose deposition within the cancer cell increases the probability of cell killing. Concerning the impact of NBTXR3 nanoparticles on the β parameter value, no effect was observed.Most normal tissues have low alpha-beta ratios suggesting that dose fractionation (daily fractions of 2 Gy) increases survival and decreases late toxicity as compared to a single equivalent dose. Tumors in contrast have high alpha-beta ratios and do not benefit from fractionation. The radiation tolerance dose is generally expressed quantitatively in terms of the biologically effective dose (BED), as defined by the LQ model. BED is the total dose required to obtain an equal biological effect for a certain endpoint, such as a given log cell kill. In presence of NBTXR3 nanoparticles, we estimated a decrease of the BED (see black arrows in Figure 8) which may be correlated with an increase of the α parameter in these cancer cell lines.

### Toward a predictive in vitro radioenhancement of NBTXR3 nanoparticles in human cancer cell lines?

Radioenhancement of NBTXR3 nanoparticles in all tested human cancer cell lines was found to increase with both NBTXR3 nanoparticle concentration and radiation dose. For the following cancer cell lines, HCT 116, NCI-H460-luc2, PANC-1, Hs913T, HT-1080, and 42-MG-BA, above a certain nanoparticle concentration and 2 Gy, equation 2 predicted an observable effect. According to equation 2, for a radiation dose (D) of 2 Gy and more, the DEF values were found to vary linearly with the dose D (i.e. the DEF value was equal to the DEF value at 2 Gy multiplied by the extra quantity of radiation dose 0.5 × X Gy). The nanoparticles concentration was dependent on the cell line. For the epithelial cancer cell lines, a minimum NBTXR3 concentration of 800 μM was necessary, whereas in the mesenchymal and glioblastoma cancer cell lines a lower nanoparticle concentrations, typically between 100 μM and 400 μM, was needed. The NBTXR3 nanoparticles cell uptake was related to some extent to the biological behavior of each cancer cell line. For instance, in the 42-MG-BA model, a marked uptake was seen in all the observed cells at the low NBTXR3 concentration of 100 μM, whereas for HCT 116 cells it was observed at 800 μM or above (see in Figure 2). Of note, *in vitro* NBTXR3 cellular uptake was observed to be lower in CAL-33 and FaDu cell lines at NBTXR3 concentration 800 μM (data not shown).

These results suggest that *in vitro*, a minimum quantity of nanoparticles in the cell – *i.e.* a minimum number of NBTXR3 nanoparticles clusters per cell – may be required to predict an observable effect, whatever the cancer cell line.

Monte Carlo calculations have demonstrated that NBTXR3 nanoparticles locally enhance the radiation dose deposit when exposed to ionizing radiation [2]. Here we show *in vitro* that activation of NBTXR3 nanoparticles from within the cancer cells by 200 kVp X-rays, triggers a biological response which seems correlated to the inherent radiosensitivity of the cancer cell line and also to the radiation dose delivered.

## Conclusion

In the panel of human cancer cell lines tested, a differential NBTXR3 nanoparticles cell uptake was observed between epithelial and mesenchymal or glioblastoma cell lines. Other characteristics such as the aggressive behavior of the cancer cells may also be considered. Besides, the clonogenic survival curves showed an increase of the α parameter, *i.e.* a decrease of the BED, with increasing NBTXR3 nanoparticles concentration.

Interestingly, while Monte Carlo calculations have already demonstrated marked radiation dose enhancement where NBTXR3 nanoparticles are, we showed *in vitro* that the biological response of the cancer cells treated with NBTXR3 nanoparticles and exposed to ionizing radiation may be anticipated from the intrinsic radiosensitivity of the cancer cell line and the radiation dose delivered. In the future, more cancer cells lines should be evaluated to confirm these promising results.

## Competing interests

All s are employees from Nanobiotix and have financial involvement with Nanobiotix, which is developing the NBTXR3 product discussed in the manuscript. JM, AP and LL are coinventors on a patent application related to the NBTXR3 material.

## Authors’ contributions

JM conceived the study, elaborated the design, carried out practical work, acquired and analyzed data and drafted the manuscript. NMA, PZ and SV carried out practical work and participated to the discussion of the data. LL contributed to conception and modified the manuscript. EB conceived the study, and modified the manuscript. AP conceived the study, participated in its design and coordination and modified the manuscript. All authors read and approved the final manuscript.

## Supplementary Material

Additional file 1 **NBTXR3 nanoparticles.** NBTXR3 nanoparticles hydrodynamic diameter and a polydispersity index (about 50 nm and 0.100, respectively) were determined by dynamic light scattering technique (Zetasizer NanoZS, Malvern Instruments Ltd, Worcestershire, UK). The surface charge of the nanoparticles in aqueous solution at pH values between 6 and 8 (about -50 mV) was estimated by zeta potential analysis (Zetasizer NanoZS). Spherical nanoparticle shape was determined using transmission electron microscopy technique (JEOL JEM 100CX operating at 100 kV, Service de Microscopie Electronique, UMR 7197, UPMC, Paris, France). Click here for file

Additional file 2 **Human cancer cell lines and culture conditions.** The HCT 116 and HT-29 cells were grown in McCoy’s 5a medium (Invitrogen, Fisher scientific, Illkirch, France), supplemented with 10% (v/v) of heat-inactivated fetal calf serum (FCS) (Invitrogen). The NCI-H460-luc2 cells were grown in Dulbecco’s Modified Eagle’s Medium (DMEM) with GlutaMAX™ medium (Invitrogen), supplemented with 10% of FCS (PAA, Velizy-Villacoublay, France). The PANC-1, HT-1080, CAL-33 and Hs913T cells were grown in DMEM with GlutaMAX^TM^ (Invitrogen), supplemented with 10% of inactivated FCS (Invitrogen). The 42-MG-BA cells were grown in DMEM with GlutaMAX^TM^ (Invitrogen) and Minimum Essential Media (MEM) (1:1) (Invitrogen), supplemented with 10% FCS (Invitrogen). The FaDu cells were grown in MEM with GlutaMAX^TM^ (Invitrogen), supplemented with 10% FCS (Invitrogen). All cells lines were kept in an incubator at 37°C under 5% CO_2_ humidified atmosphere. Click here for file
