# Supplementary material for: Hafnium oxide nanoparticles: toward an in vitro predictive biological effect?
Source: Radiat Oncol. 2014 Jun 30;9:150. doi: 10.1186/1748-717X-9-150 (PMC4104401; doi:10.1186/1748-717X-9-150)
Supplement: Additional file 1 — NBTXR3 nanoparticles. NBTXR3 nanoparticles hydrodynamic diameter and a polydispersity index (about 50 nm and 0.100, respectively) were determined by dynamic light scattering technique (Zetasizer NanoZS, Malvern Instruments Ltd, Worcestershire, UK). The surface charge of the nanoparticles in aqueous solution at pH values between 6 and 8 (about -50 mV) was estimated by zeta potential analysis (Zetasizer NanoZS). Spherical nanoparticle shape was determined using transmission electron microscopy technique (JEOL JEM 100CX operating at 100 kV, Service de Microscopie Electronique, UMR 7197, UPMC, Paris, France). [file 1748-717X-9-150-S1.doc]

**Additional File 1: NBTXR3 nanoparticles**

NBTXR3 nanoparticles hydrodynamic diameter and a polydispersity index (about 50nm and 0.100, respectively) were determined by dynamic light scattering technique (Zetasizer NanoZS, Malvern Instruments Ltd, Worcestershire, UK). The surface charge of the nanoparticles in aqueous solution at pH values between 6 and 8 (about -50mV) was estimated by zeta potential analysis (Zetasizer NanoZS). Spherical nanoparticle shape was determined using transmission electron microscopy technique (JEOL JEM 100CX operating at 100 kV, Service de Microscopie Electronique, UMR 7197, UPMC, Paris, France).
