# Supplementary material for: Hafnium oxide nanoparticles: toward an in vitro predictive biological effect?
Source: Radiat Oncol. 2014 Jun 30;9:150. doi: 10.1186/1748-717X-9-150 (PMC4104401; doi:10.1186/1748-717X-9-150)
Supplement: Additional file 2 — Human cancer cell lines and culture conditions. The HCT 116 and HT-29 cells were grown in McCoy’s 5a medium (Invitrogen, Fisher scientific, Illkirch, France), supplemented with 10% (v/v) of heat-inactivated fetal calf serum (FCS) (Invitrogen). The NCI-H460-luc2 cells were grown in Dulbecco’s Modified Eagle’s Medium (DMEM) with GlutaMAX™ medium (Invitrogen), supplemented with 10% of FCS (PAA, Velizy-Villacoublay, France). The PANC-1, HT-1080, CAL-33 and Hs913T cells were grown in DMEM with GlutaMAXTM (Invitrogen), supplemented with 10% of inactivated FCS (Invitrogen). The 42-MG-BA cells were grown in DMEM with GlutaMAXTM (Invitrogen) and Minimum Essential Media (MEM) (1:1) (Invitrogen), supplemented with 10% FCS (Invitrogen). The FaDu cells were grown in MEM with GlutaMAXTM (Invitrogen), supplemented with 10% FCS (Invitrogen). All cells lines were kept in an incubator at 37°C under 5% CO2 humidified atmosphere. [file 1748-717X-9-150-S2.doc]

**Additional File 2: Human cancer cell lines and culture conditions**

The HCT 116 and HT-29 cells were grown in McCoy’s 5a medium (Invitrogen, Fisher scientific, Illkirch, France), supplemented with 10% (v/v) of heat-inactivated fetal calf serum (FCS) (Invitrogen). The NCI-H460-luc2 cells were grown in Dulbecco’s Modified Eagle’s Medium (DMEM) with GlutaMAX^TM^ medium (Invitrogen), supplemented with 10 % of FCS (PAA, Velizy-Villacoublay, France). The PANC-1, HT1080, CAL-33 and Hs913T cells were grown in DMEM with GlutaMAX^TM^ (Invitrogen), supplemented with 10% of inactivated FCS (Invitrogen). The 42-MG-BA cells were grown in DMEM with GlutaMAX^TM^ (Invitrogen) and Minimum Essential Media (MEM) (1:1) (Invitrogen), supplemented with 10% FCS (Invitrogen). The FaDu cells were grown in MEM with GlutaMAX^TM^ (Invitrogen), supplemented with 10% FCS (Invitrogen). All cells lines were kept in an incubator at 37°C under 5% CO_2_ humidified atmosphere.
